# Long‐term outcomes in patients with intracorporeal robot‐assisted pyramid neobladder

**DOI:** 10.1002/bco2.70062

**Published:** 2025-08-11

**Authors:** Elizabeth Day, Pratham Upadhyay, Raashi Padhiyar, Lazaros Tzelves, Bernadett Szabados, Anthony Ta, Ashwin Sridhar, John Kelly

**Affiliations:** ^1^ Department of Urology University College London Hospital London UK; ^2^ Medical School University College London London UK; ^3^ 2nd Department of Urology, Sismanogleio Hospital National and Kapodistrian University of Athens Greece; ^4^ Barts Experimental Cancer Medicine Centre, Barts Cancer Institute Queen Mary University of London London UK; ^5^ Department of Urology St Vincent Hospital Melbourne Australia

**Keywords:** bladder cancer, neobladder, reconstructive urology, robotic cystectomy, urinary diversion

## Abstract

**Objectives:**

A range of techniques have been described for robotic‐assisted intracorporeal neobladder construction. The pyramid neobladder has now been performed for over 10 years. We now describe the long‐term outcomes, including the impact of function preservation through nerve and prostate capsule sparing in the male population.

**Subjects/Patients and Methods:**

All patients who underwent pyramid neobladder construction between January 2015 and December 2023 at the University College London Hospital (UK) were reviewed. Patients were selected for function preservation based on baseline sexual function/wishes, PSA ± multiparametric prostate MRI. Selected oncological, functional and patient reported outcomes were assessed.

**Results:**

A total of 71 patients were included; 87% (61/71) were men. About 65% (40/61) underwent either nerve‐ or prostate capsule‐sparing surgery. Median follow‐up was 57.8 months (IQR 48.3). In male patients, there was no difference between the 12‐ and 24‐month cancer specific and overall survival rates between the function sparing groups. There were no positive prostate cancer margins. Daytime continence was 75% (39/52) and 67% (6/9), and nighttime was 17% (9/52) and 11% (1/9), in men and women, respectively. There was no significant difference between the male function sparing groups (*p* < 0.342). About 94% of men (29/31) were sexually active before surgery, falling to 55% (17/31) after, with 76% (13/17) using treatment for erectile dysfunction. The median change in SHIM score was 4.5 (SD 5.3) in prostate capsule‐sparing, 6 (SD 7.7) in nerve‐sparing and 17 (SD 2.8) in standard groups.

About 45% (30/66) of patients had a significant reduction in eGFR (>10 mL/min/1.73m^2^). Uretero–ileal strictures were confirmed in 4.2% (3/71). About 28% (19/67) of patients reported recurrent UTIs and 7% (5/71) reported neobladder rupture.

**Conclusion:**

Sexual function had the largest impact on quality of life and may be improved with function‐sparing techniques. The burden of additional complications including neobladder rupture and urinary tract infections was also highlighted.

## 1 INTRODUCTION

Robot‐assisted radical cystectomy (RARC) with intracorporeal urinary diversion was first described in 2003.^1^ Intracorporeal reconstruction has grown in popularity with the International Robotic Cystectomy Consortium database reporting a rise in intracorporeal neobladder construction from 0% in 2005 to 23% in 2018.^2^ Techniques have also evolved with nine detailed in a recently published atlas.^3^ We described a 50‐cm detubularised folded neobladder which evolved with an afferent limb to accommodate high ureteric division and re‐implantation into the limb.^4^ The pyramid neobladder has now been performed for over 10 years.^4^

In addition to the neobladder construction, surgical techniques can be modified with the aim to preserve urinary and sexual function. In the male population, this includes preservation of the neurovascular bundles surrounding the prostate or preservation of the prostate capsule with enucleation of the transitional zone with the bladder specimen.^5^ In the female population, function preservation includes preservation of vaginal length, the uterus and ovaries.

We present long‐term outcomes of intracorporeally constructed pyramid neobladder and assess the impact of function sparing techniques.

## 2 PATIENTS AND METHODS

Between January 2015 and December 2023, 71 patients, of 677 cystectomies, received a pyramid neobladder. Pre‐operative, operative and follow‐up data were collected from electronic patient records. This study was logged with the Institutional Review Board as a clinical service evaluation (Ref: UROONC202404).

### 2.1 Patient selection and technique

A multidisciplinary planning meeting recommended the type of urinary diversion and optimal function preserving approach based on imaging (CT ± MRI), histopathology and baseline sexual function. Surgery was performed by four high volume surgeons. The neobladder technique has been reported previously.^4^ Nerve‐sparing and prostate capsule‐sparing techniques and selection have also been described previously.^5^, ^6^ Full organ‐sparing approach in female cystectomy encompassed preservation of the uterus, ovaries as well minimal impact of a vaginal length.^6^

### 2.2 Oncological outcomes

The positive surgical margin (PSM) rate, recurrence free, 12‐month and 24‐month cancer specific and overall survival rates are reported. Incidental prostate cancer and prostate cancer PSM were recorded separately.

### 2.3 Functional outcomes

Urinary continence was assessed at a minimum of 12 months. Continence (pad number) was recorded for daytime and nighttime. Hypercontinence was defined as voiding dependent on intermittent self‐catheterisation (ISC).

Renal functions at baseline and at last follow‐up were recorded and changes grouped into CKD groups.^7^ A reduction in eGFR of more than 10 mL/min/1.73m^2^ was consider significant.^8^ The cause of hydronephrosis on imaging was differentiated as relating to stricture, disease progression, poor bladder emptying or unknown nonobstructive cause. Dynamic imaging with MAG3 renogram and/or antegrade nephrostogram as well as video‐urodynamics was used in this assessment. Urolithiasis was identified on surveillance scans or on symptomatic presentation. Neobladder rupture was identified on imaging and/or patient report when presentation was outside our centre.

### 2.4 Patient reported outcomes

All patients were asked to completed the EORTC BLM‐30 health related quality of life questionnaire.^9^ For sexual function, male patients completed a SHIM score,^10^ and any treatment for erectile dysfunction was recorded, and female patients completed the FSFI‐6 questionnaire.^11^ Patients were also asked to report recurrent urinary tract infection (rUTIs) and commencement of prophylactic treatment. Patients were contacted three times before being labelled as nonresponders.

### 2.5 Statistical analysis

Continuous outcomes are described as medians/ranges and compared with Wilcoxon‐rank sum test. Categorical outcomes are described as numbers and proportions and compared using Chi‐square test.

The BLM‐30 module was analysed according to the scoring manual.^12^ All scores were linearly transformed on a 0–100 scale; a score of 100 indicated worst quality of life. For all PROMS questionnaires, mean scores with standard deviation were calculated.

Significance threshold was set at *p* < 0.05 although the small groups sizes are acknowledged as a key limitation.

## 3 RESULTS

### 3.1 Patient and disease characteristics (Table 1)

**TABLE 1:** Oncological characteristics and outcomes.

	Total	Female organ sparing	Female standard	Male capsule sparing	Male nerve sparing	Male standard
Total	71	6	3	10	30	22
Presurgical treatment
BCG failure	9 (12.7%)	0	1	1	5	2
Neo‐adjuvant systemic therapy	32 (45.0%)	1	1	3	15	12
Clinical stage
NMIBC	29 (40.8%)	2	1	5	13	8
T2	39 (54.9%)	4	1	4	16	14
T3/4	3 (4.2%)	0	1	1	1	0
Nx	1 (1.4%)	0	1	0	0	0
N0	67 (94.4%)	6	3	9	28	21
N+	3 (4.2%)	0	0	1	2	0
Pathological stage*
T0	26 (36.6%)	1	1	1	13	10
NMIBC	24 (33.8%)	2	1	5	8	8
T2	6 (8.4%)	0	0	1	4	1
T3/4	15 (21.2%)	3	1	3	5	3
Nx	3 (4.3%)	2	0	0	1	0
N0	58 (81.7%)	4	2	9	24	19
N+	10 (14.1%)	0	1	1	5	3
Positive surgical margin	3 (4.3%)	0	0	1	1	1
Prostate cancer	‐	‐	‐	1	10	6
Histology type**
Urothelial	62 (87.3%)	1	2	8	30	21
Subtype histology	12 (16.9%)	1	0	4	7	0
Squamous carcinoma	8 (11.3%)	4	1	2	0	1
Adenocarcinoma	1 (1.4%)	1	0	0	0	0
Oncological outcomes
12‐month cancer specific survival rate	92.7%	100%	100%	90%	90%	95%
24‐month cancer specific survival rate	88.5%	100%	100%	80%	84.6%	94%
12‐month overall survival rate	90%	100%	100%	90%	90%	90%
24‐month overall survival	84.3%	100%	100%	80%	84.6%	83%

*Final pathological stage at cystectomy.

** On TURBT and/or cystectomy histology.

A total of 71 patients were included; the median age was 58 years (IQR = 13) years with 13% (9/71) women, mean age of 51 years and 87% (62/71) men and mean age of 58 years. In the female cohort, 66% (6/9) underwent organ‐sparing. In the male cohort, 65% (40/62) underwent function sparing in the form of prostate capsule‐sparing (16%, 10/62) and nerve‐sparing (48%, 30/62).

### 3.2 Oncological outcomes (Table 1)

The disease characteristics and oncological outcomes are provided in Table 1. Overall, there was no difference between the 12‐ and 24‐month cancer specific and overall survival rates between the function sparing groups.

There were three positive margins: two cases of carcinoma in situ at the ureteric margin, with neither patient experiencing recurrence, and one case of circumferential margin which progressed with loco‐regional recurrence.

In the capsule‐sparing group, 10% (1/10) of patients had Gleason 3+3 prostate cancer. In the nerve‐sparing group, 50% (15/30) of patients had prostate cancer (G3+3, 8 patients and G3+4, 7 patients). In the standard group, 27% (6/22) of patients had prostate cancer (G3+3, 4 patients and G4+3, 2 patients). There were no positive prostate cancer margins.

### 3.3 Functional outcomes (Table 2)

**TABLE 2:** Urinary and renal function outcomes.

	Total	Female organ sparing	Female standard	Male capsule sparing	Male nerve sparing	Male standard
Total	71	6	3	10	30	22
Median age	58 (13)	48 (11)	63 (‐)	50 (8)	57 (9)	61 (7)
Urinary function^a^ (completeness = 86%)						
Daytime continence (0 pads)	44 (72%)	4	1	6	23	10
Daytime social continence	49 (80%)	4	1	8	24	12
Daytime uro‐sheath	‐	‐	‐	0	0	1
Nighttime continence (0 pads)	10 (16%)	1	0	3	5	1
Night time social continence	19 (31%)	1	0	5	7	6
Nighttime uro‐sheath	‐	‐	‐	3	18	7
Hypercontinence	6 (10%)	1	1	1	3	0
Renal function and stricture (completeness = 93%)						
Decline by eGFR > 10 mL/min/1.73m^2^	30 (45%)	1	2	4	11	12
Decline into CKD Stage 3/4^b^	13 (21%)	0	1	3	6	3
Uretero–ileal stricture	3 (4.3%)	0	0	0	2	1

^a^ In all patients with a minimum of 12‐month follow‐up.

^b^ Excluding four patients whose baseline renal function was CKD Stage 3.

#### 3.3.1 Male urinary function

Continence data were available for 52 men. Using a strict definition (0 pads), 17% (9/52) were continent both day and night and 75% (39/52) were continent during the day. Daytime continence following capsule‐sparing was 60% (6/10), nerve‐sparing was 77% (23/30) and standard approach was 46% (10/22). There was no significant difference between the groups for daytime (*p* < 0.17) or nighttime continence (*p* < 0.16).

Daytime social continence (1 pad or fewer) following capsule‐sparing was 80% (8/10), for nerve‐sparing was 80% (24/30) and standard approach was 55% (12/22). There was no significant difference between the groups for daytime (*p* < 0.11) or nighttime continence (*p* < 0.27).

Of the men that were not socially continent, 33% (1/3) used a uro‐sheath during the day and 85% (28/33) used one at night. One man, of the nonfunction sparing group, had undergone implantation of an artificial urinary sphincter for incontinence.

Hypercontinence was reported for 7% (4/57) men; three men had undergone a nerve‐sparing, and one man capsule‐sparing.

Neobladder rupture occurred in 8% (5/62) men between 6 months to 6 years after cystectomy. All ruptures were spontaneous, that is, in the absence of trauma/surgery, but one patient had received adjuvant radiotherapy. Two patients underwent a laparotomy and repair, and three patients were managed conservatively with drainage. No patient experienced subsequent rupture.

#### 3.3.2 Female urinary function

Continence data were available for all nine women. Using the strict definition (0 pad), one woman was continent at day and night; she was also hypercontinent. About 55% (5/9) of women were continent during the day and 11.1% (1/9) at night.Daytime continence was not significantly associated with organ sparing (*p* < 0.342). Social continence rates were the same as continence rates using the strict definition. No women underwent subsequent surgery for incontinence, and there were no cases of neobladder rupture.

#### 3.3.3 Male sexual function

Sexual function data were available for 31 men, of whom 94% (29/31) were sexually active before surgery and 55% (17/31) after surgery, with 76% (13/17) using treatment for erectile dysfunction. One patient had received a penile implant; the remainder used a vacuum device, oral PDE5 inhibitors, injectable and/or intra‐urethral therapies. About 77% (10/13) men reported that the treatment for erectile dysfunction was effective. The median change in SHIM score before and after surgery was 4.5 points (SD 5.3) in the prostate capsule‐sparing group, 6 points (SD 7.7) in the nerve sparing group and 17 points (SD 2.8) in standard group. The statistical significance of differences between the function and nonfunction sparing groups could not be assessed due to the small number (4) of sexually active men responding in the standard group.

#### 3.3.4 Female sexual function

Data were available for two women. One woman was sexually active and reported a FFSI‐6 score of 20, and the other woman was not sexually active, reporting a score of 3.

#### 3.3.5 Renal function and stricture rate

Renal function data were available for 66 patients. Baseline renal function was in CKD groups 1 or 2 for 94% (62/66) of patients with the remaining in CKD group 3a (6.0% [4/66]).

A significant reduction in eGFR, defined as more than 10 mL/min/1.73m^2^, was seen in 45% (30/66) of patients. A fall from CKD groups 1 or 2 into groups 3 or 4 was seen in 21% (13/62) of patients. For the patients with a baseline renal function in CKD group 3a, none declined by more than 10 mL/min/1.73m^2^ in the follow‐up period.

Uretero–ileal stricture was confirmed in three male patients (4.2%). One patient underwent a robotic‐assisted nephroureterectomy as the kidney was nonfunctioning kidney at the point of diagnosis, 10 months after cystectomy. The other strictures were diagnosed at 5 and 2 months and were managed with robotic‐assisted ureteric re‐implantation.

An additional 14 patients had hydronephrosis (9 bilateral, 5 unilateral) on imaging, 5 related to cancer progression, 2 to poor bladder emptying/reflux and the remaining 7 were nonobstructive with no clear cause.

#### 3.3.6 Urolithiasis

No patients developed bladder calculus. Upper tract stones were diagnosed in 6% (4/71) of patients. One patient underwent ESWL, one required a long‐term nephrostomy and the remaining underwent surveillance.

### 3.4 Patient report outcomes (Table 3)

**TABLE 3:** Patient report outcomes.

	Total	Female organ sparing	Female standard	Male capsule sparing	Male nerve sparing	Male standard
Total	71	6	3	10	30	22
UTIs (completeness = 94%)						
Recurrent UTIs reported	19 (28%)	4	1	1	6	7
Use of prophylactic measures for UTIs	9 (12%)	3	1	1	2	2
Male sexual function (completeness = 44%)						
Response the sexual function questions	31	‐	‐	4	15	12
Sexually active before surgery	29 (93%)	‐	‐	4	13	12
ED treatment before surgery	5 (16%)	‐	‐	1	3	1
Sexually active after surgery	17 (55%)	‐	‐	4	8	5
ED treatment after surgery	13 (42%)	‐	‐	2	9	2
ED treatment effective^a^ after surgery	10 (77%)^b^	‐	‐	1	5	1
Response to SHIM score	15	‐	‐	7	4	4
Median SHIM score before	24 (4.3)	‐	‐	22.5 (3.2)	24 (5.7)	23.5 (1.3)
Median SHIM score after	11 (6.4)	‐	‐	18 (8.4)	12 (5.7)	6.5 (2.4)
Median change in SHIM score	8 (7.1)	‐	‐	4.5 (5.3)	6 (7.7)	17 (2.8)
Female sexual function (completeness = 22%)						
Response to FFSI‐6	2	2	0	‐	‐	‐
FFSI score	2	20.3	‐	‐	‐	‐
Sexually active before	2	2	‐	‐	‐	‐
Sexually active after	1	1	‐	‐	‐	‐

^a^ Patient perceived,

^b^ Of those that used ED treatment.

#### 3.4.1 Urinary tract infections

Patient reported rUTIs were observed in 28.4% (19/67) of cases. rUTI necessitating prophylaxis was reported by 11.9% (9/67) of patients and was most prevalent in females, with 57% (4/7) using prophylaxis in comparison to 8% (5/60) of men. Prophylactic management included low dose daily antibiotics and nonantibiotic measures such as methenamine hippurate (four patients), D‐mannose (two patients) and cranberry supplements (one patient).

### 3.5 Health‐related quality of life outcomes (Figure 1)

**FIGURE 1:**
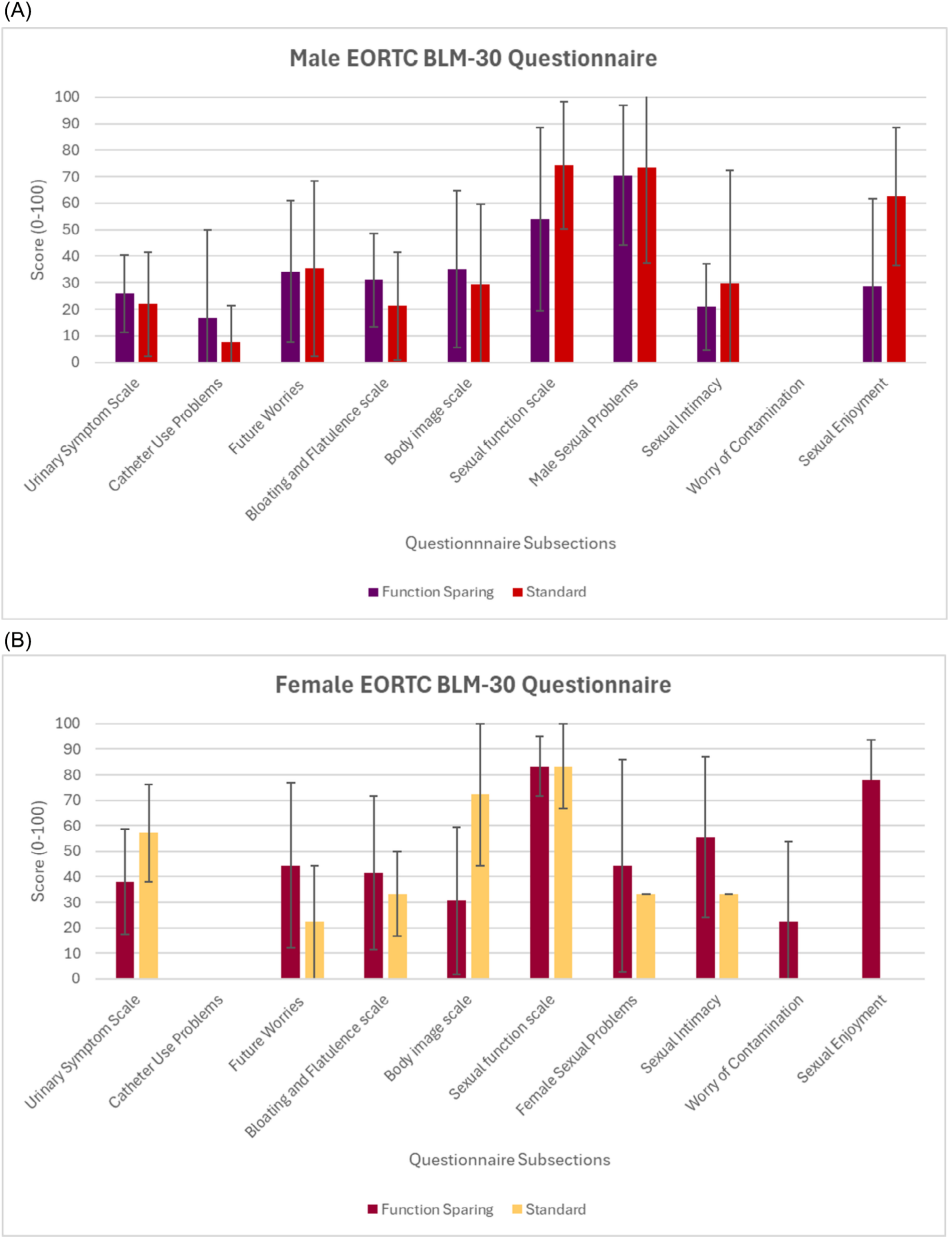
Patient reported outcomes: Quality of life by sex and function sparing. (A) Male patients. (B) Female patients. EORTC BLM‐30 by sex and function sparing. Scores have been linearly transformed on a scale from 0 to 100, with a higher score representing worse outcomes. The bar graph illustrates mean scores, with error bars representing standard deviation.

Of the 59 patients alive, 32 patients (54.2%), 7 women and 25 men, responded to the BLM 30 module questionnaire. Sexual function domains appeared to have the greatest impact on quality of life followed by future worries and bloating and flatulence. Urinary symptoms scored higher for female than male patients, but statistical comparison between the sexes and between the function sparing groups was not possible.

## 4 DISCUSSION

We present a large series of robotic intracorporeal neobladder formation, specifically using the pyramid neobladder, with long‐term follow‐up and report on functional outcomes.

The overall daytime and nighttime continence (0 pads) rates were 72% and 16%, respectively. Studies assessing a range of neobladder techniques have reported 1‐year daytime continence (0–1 pad definitions) ranging from 86% to 90% and nighttime continence at 66%–75%.^13^, ^14^, ^15^ The daytime rates observed for the pyramid neobladder are therefore comparable, but the nighttime rates are lower and this may be due to the use of a strict definition of continence of 0 pads/day. This definition was adopted due to reports of the significant difference in quality of life between patients using 0 and 1 pads.^16^, ^17^ There was no demonstrable benefit of function sparing approaches for urinary continence and was also the conclusion in a recent meta‐analysis of capsule‐sparing cystectomy.^5^

The overall hypercontinence rate was 10%. Other series report ISC rates ranging from 0% to 33% but vary in their recommendations for what post‐void residual are used to recommend ISC so comparison is difficult.^18^, ^19^ Rates of neobladder rupture are also difficult to compare as they not reported in contemporary robotic series. In one large open series, the rate of spontaneous rupture was less than 0.1%.^20^ The rate seen in this series is higher at 7%, and adjuvant radiotherapy and patient compliance were identified as contributory factors.

Over half of men responding to the sexual function survey were sexually active post cystectomy, with 70% of these men requiring treatment for erectile dysfunction. The impact of this decline in function is seen in the BLM‐30 questionnaire, where sexual function domains had the largest impact on quality of life. In this series, there is a clear trend for improved sexual function in patients who received capsule‐ and nerve‐sparing compared to the standard approach. A recent meta‐analysis described improved sexual function with capsule‐sparing, and the smaller median changes in SHIM score in this series in the function sparing groups support this finding.^5^ We also report on the oncological outcomes of function sparing, with no prostate cancer or urothelial positive margins seen. This further supports the feasibility and safety of these approaches. Case selection is key, and the prostate cancer detection rate in the capsule‐sparing group was lower than in nerve‐sparing consistent with appropriate selection using PSA and multiparametric MRI.

The number of female patients in this series was low consistent with other reports and highlights the relevance of meta‐analyses to understand the functional impact organ‐sparing; A recent metanalysis concluded that there is benefit for urinary continence, but further evidence is needed for sexual function.^21^

A significant decline in renal function was seen in 45% of patients which is comparable to other studies.^8^ About 14.5% of patients fell into CKD group 3b or more from a baseline of groups 1 or 2. This is a significant threshold as it may limit eligibility for adjuvant treatment in the future. However, this finding is not specific to a continent diversion and is likely to be multifactorial, relating to medical comorbidities and oncological treatment, as there was no demonstrable association with the presence of hydronephrosis in this series.^22^

rUTIs were reported by 28% of patients, with approximately half of these patients utilising prophylactic prevention strategies. The burden in the female cohort was high, with 57% of women using, or had used, prophylaxis for rUTIs. There is no evidence on the optimal prevention strategies, and this is a key area of future work to improve functional outcomes.

The study is limited by its retrospective nature and, as a result, by the variation in the level of missing data between the outcome measures. Also, as outcomes were recorded at last follow‐up, patients at different stages of recovery were amalgamated. However, all patients had a minimum follow‐up of 12 months such that the initial recovery had been overcome. Small numbers in groups also limited the study's conclusions; the small numbers of sexually active patients particularly limited the data on sexual function. Additionally, the small number of women, a common problem in series of this kind, has created a gender bias as regards available outcome data.

## 5 CONCLUSION

Overall, this study reports on long‐term functional outcomes following intracorporeal robotic construction of a pyramid neobladder. Continence outcomes are comparable to other neobladder types but do not appear to be improved by function sparing. Sexual function has the largest impact on quality of life, and consistent with other series, we show a benefit for a capsule‐sparing and demonstrate its oncological safety in carefully selected cases.

## AUTHOR CONTRIBUTIONS

*Conception and design*: Elizabeth Day and John Kelly. *Acquisition of data*: Elizabeth Day, Pratham Upadhyay, Raashi Padhiyar, Anthony Ta and Lazaros Tzelves. *Analysis and interpretation of data*: Elizabeth Day, Pratham Upadhyay, Bernadett Szabados and Lazaros Tzelves. *Drafting of manuscript*: Elizabeth Day. *Critical revision*: John Kelly and BES. *Statistical analysis*: Pratham Upadhyay and Lazaros Tzelves. *Supervision*: John Kelly.

## CONFLICT OF INTEREST STATEMENT

The authors report no other relevant conflicts of interest.
